# IL-17A stimulates the production of inflammatory mediators via Erk1/2, p38 MAPK, PI3K/Akt, and NF-κB pathways in ARPE-19 cells

**Published:** 2011-11-23

**Authors:** Ying Chen, Aize Kijlstra, Yuanyuan Chen, Peizeng Yang

**Affiliations:** 1 Zhongshan Ophthalmic Center, Sun Yat-sen University, Guangzhou, People’s Republic of China; 2 The First Affiliated Hospital of Chongqing Medical University, Chongqing Key Laboratory of Ophthalmology, Chongqing Eye Institute, Chongqing, People’s Republic of China; 3 Eye Research Institute Maastricht, Department of Ophthalmology, University Hospital Maastricht, Maastricht, The Netherlands

## Abstract

**Purpose:**

To investigate the signaling pathways involved in interleukin (IL)-17A -mediated production of interleukin 8 (CXCL8), chemokine (C-C motif) ligand 2 (*CCL2),* and interleukin 6 (IL-6) by ARPE-19 cells, a spontaneously arisen cell line of retinal pigment epithelium (RPE).

**Methods:**

Flow cytometry analysis and western blot were used to detect the phosphorylation of extracellular signal-regulated kinases 1/2 (Erk1/2), p38 mitogen activated protein kinase (MAPK) and protein kinase B (PKB; Akt) in ARPE-19 cells stimulated with IL-17A. These cells were further pretreated with a series of kinase inhibitors and followed by incubation with IL-17A. CXCL8, CCL2, and IL-6 in the supernatant were quantified by enzyme-linked immunosorbent assay (ELISA).

**Results:**

Coculture of ARPE-19 cells with IL-17A resulted in significant increases in Erk1/2, p38 MAPK, and Akt phosphorylation. Inhibition of p38MAPK, phosphoinositide 3-kinase (PI3K)-Akt and nuclear factor-kappaB (NF-κB), with the inhibitors SB203580, LY294002 and pyrrolydine dithiocarbamate (PDTC) respectively, reduced IL-17 (100 ng/ml) mediated production of CXCL8, CCL2, and IL-6 in a concentration dependent manner. Inhibition of Erk1/2 with PD98059 decreased the expression of the tested three inflammatory mediators when using low doses of IL-17A (0–10 ng/ml) but not at higher concentrations.

**Conclusions:**

IL-17A-induced production of inflammatory mediators by ARPE-19 cells involves Erk1/2, p38MAPK, PI3K-Akt and NF-κB pathways.

## Introduction

Uveitis is a common intraocular inflammatory disease. Recent studies have shown that helper T lymphocyte (Th)17 cells are implicated in the pathogenesis of this serious intraocular disorder [1,2]. They have been identified as a subset of T-helper lymphocytes characterized by predominantly producing interleukin (IL)-17A [3,4]. Growing evidence suggests that Th17 cells trigger inflammatory responses primarily via IL-17A [5].

A recent study showed an increased expression of *IL-17A* mRNA in the retina of mice with experimental autoimmune uveoretinitis (EAU), a classical model for human autoimmune uveitis [1]. IL-17A protein was furthermore found to be highly expressed by peripheral blood mononuclear cells (PBMCs) from uveitis patients [6,7]. IL-17A is a proinflammatory cytokine which is reflected by its ability to promote a variety of cells to produce chemokines and proinflammatory cytokines including interleukin-8 (CXCL8), CCL2, and IL-6 [8].

The neuroectodermally-derived retinal pigment epithelium (RPE), strategically positioned at the blood-retinal barrier, is considered to play an important role in posterior ocular inflammation due to its ability to secrete several inflammatory mediators [9]. CXCL8, CCL2, and IL-6 are three major inflammatory mediators produced by RPE cells in response to various stimuli [9]. Several studies have shown that these mediators are involved in the pathogenesis of uveitis [10-12]. CXCL8 is a chemoattractant and activator of neutrophils, whereas CCL2 is a chemoattractant and activator for lymphocytes and monocytes. These two chemokines mediate neutrophil, lymphocyte and monocyte/macrophage infiltration into tissues. IL-6 is a pleiotropic proinflammatory cytokine. The overexpression of IL-6 may intensify the local immune and inflammatory response.

In a previous study we showed that IL-17A is a potent stimulus for CXCL8, CCL2, and IL-6 secretion by ARPE-19 cells [13], the spontaneously arisen human RPE-derived cell line which has been extensively used in the past decades to investigate the role of this cell layer in the pathogenesis of ocular posterior diseases including uveitis. It has been reported that activation of extracellular signal-regulated kinases 1/2 (Erk1/2), p38 mitogen activated protein kinase (MAPK), and phosphoinositide 3-kinase (PI3K)-Akt is involved in the IL-17A induced response of certain cell types [14-17]. However, the signaling events leading to CXCL8, CCL2, and IL-6 protein expression by IL-17A-induced ARPE-19 cells have not yet been characterized. In this study, we therefore investigated the role of Erk1/2, p38 MAPK, and PI3K-Akt in IL-17A-induced CXCL8, CCL2, and IL-6 protein production.

## Methods

### Cell culture

Human ARPE-19 cells were obtained from the American type culture collection (ATCC, Manassas, VA), and cells between passages 16 and 20 were used for experiments. The cells were cultured in Dulbecco’s modified Eagle medium/F12(DMEM/F12 (Invitrogen, Beijing, China) with 10% fetal bovine serum (FBS, Invitrogen, Carlsbad, CA), 100 U/ml penicillin, and 100 μg/ml streptomycin in a humidified incubator at 37 °C in 5% CO_2_. The cells were passed every 4 to 5 days by trypsinization and were seeded into Corning flasks (Corning, Lowell, MA) at 1.2×10^6^ cells/flask, resulting in completely confluent (≈1.2×10^6^ cells/flask) cultures in 4 days.

### Flow cytometry analysis

Flow cytometry analysis was used to detect the activation state of signaling pathway kinases in ARPE-19 cells. Confluent ARPE-19 cells maintained in serum-free medium for 24 h were cultured with or without 100 ng/ml IL-17A at 37 °C in 5% CO_2_ for the detection of phospho-Erk1/2, p38, and Akt, respectively. We conducted simultaneous staining of ARPE-19 cells for intracellular phosphorylated Erk1/2, p38, and Akt proteins according to the protocol recommended by Cell Signaling Technology (Cell Signaling Technology, Beverly, MA). Briefly, ARPE-19 cells were fixed in 4% formaldehyde for 10 min at room temperature and permeabilized in methanol for 30 min on ice. We used the phospho-specific Abs anti-phospho-Erk1/2-PE, anti-phospho-p38MAPK-Alexa Fluor 488 and anti-phospho-Akt-Alexa Fluor 488 for intracellular staining (Cell Signaling Technology). Isotype-matched irrelevant Abs were used as controls. Phosphorylation of the three proteins for both unstimulated and stimulated ARPE-19 cells was evaluated by flow cytometry and expressed as mean fluorescence intensity (MFI). All experiments were repeated three times.

### Western blot

ARPE-19 cells were serum starved in DMEM/F12 without FBS for 24 h, then treated with or without 100 ng/ml IL-17A for 7, 15, or 30 min. The cells were subsequently rinsed with ice-cold PBS and lysed with lysis buffer containing 50 mM Tris-HCl (pH 7.4), 150 mM NaCl, 1% Triton X-100, 1% sodium deoxycholate, 0.1% SDS, 2 mM EDTA, and 100 μM phenylmethylsulfonylfluoride. The cell lysate was centrifuged and the supernatant was collected. Protein concentration was determined with a protein assay (Bio-Rad, Richmond, CA). Laemmli gel loading buffer was added to the lysate and boiled for 7 min, after which proteins were separated on an SDS-polyacrylamide gel. Proteins were transferred to polyvinylidene difluoride membranes (Millipore, Bedford, MA), blocked by 5% skim milk at 37 °C for 2 h, and incubated with the primary phosphorylated or total antibodies against Erk1/2, p38MAPK, and Akt (Cell Signaling Technology) at 4 °C for 16 h, followed by a horseradish peroxidase-conjugated secondary antibody at 37 °C for 1 h. The membranes were further developed using a chemiluminescent detection kit (Cell Signaling Technology). Each stimulation experiment was repeated three times.

### Enzyme-linked immunosorbent assay (ELISA)

ARPE-19 cells were maintained in DMEM/F12 medium containing 10% FBS for 4 days to become confluent. Before treatment with signaling inhibitors, cells were serum-starved for 24 h in DMEM/F12 without FBS. ARPE-19 cells were pretreated with or without an inhibitor to Erk1/2 (PD98059 at 50, 25, and 10 μM), p38MAPK (SB203580 at 25, 10, and 1 μΜ), PI3K (LY29400 at 25, 10, and 1 μΜ) or NF-κB (PDTC at 50, 25, and 10 μM; all from Sigma-Aldrich, St. Louis, MO) for 2 h, followed by incubation with or without recombinant IL-17A (R&D Systems, Minneapolis, MN) for 24 h. The supernatants were collected and centrifuged to remove particulates and stored at −70 °C until analysis. CXCL8, CCL2, and IL-6 were measured using human commercially available ELISA development kits (Duoset; R&D Systems). Each stimulation experiment was repeated four times.

### Statistical analyses

All data are expressed as means±SD. Statistical significance of changes was determined by the Student’s *t*-test. A p<0.05 was considered to be statistically significant for all experiments.

## Results

### Effect of IL-17A on Erk1/2, p38MAPK, and Akt phosphorylation

**Figure 1:**
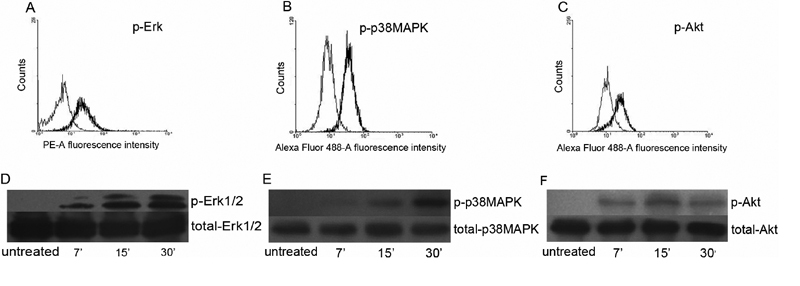
Effects of IL-17A on phosphorylation of Erk1/2, p38MAPK, or Akt in ARPE-19 cells. ARPE-19 cells were incubated without or with 100 ng/ml IL-17A whereafter the degree of phosphorylation of Erk1/2, p38 and Akt was measured. The amounts of intracellular phosphorylated signaling molecules in 10,000 permeabilized ARPE-19 cells were measured by flow cytometry. A representative example and MFI result expressed as the mean±SD of three independent experiments are shown for the phosphorylation of Erk1/2 (20 min stimulation; **A**), p38 MAPK (20 min stimulation; **B**), and Akt (20 min stimulation; **C**) in response to IL-17A (thick line histograms). Thin line histograms indicate basal phosphorylation in the absence of IL-17A. Western blot analysis of phosphorylated Erk1/2, p38MAPK or Akt was performed on ARPE-19 cells stimulated with or without 100 ng/ml of IL-17A for the indicated time periods. Results are representative of three separate experiments.

To investigate the early molecular mechanisms whereby IL-17A stimulates the production of CXCL8, CCL2, and IL-6, ARPE-19 cells were incubated for 10 or 20 min with 100 ng/ml IL-17A. The level of phosphorylated Erk1/2, p38MAPK, and Akt was evaluated by measuring mean fluorescence intensity (MFI) with flow cytometry. The result revealed that the MFI of phosphorylated Erk1/2, p38MAPK and Akt significantly increased in ARPE-19 cells stimulated by 100 ng/ml IL-17A as compared to unstimulated cells. Examples of phosphorylated specific intracellular staining are shown in Figure 1A-C. Additionally, western blot analysis was performed to verify the activation of these protein kinases. The samples were immunoblotted with antibodies against the phosphorylated form of Erk1/2, p38MAPK, and Akt. As shown in Figure 1D-F, IL-17A increased the phosphorylation of Erk1/2 and p38 MAPK by 7 min and remained elevated up to 30 min in these experiments. The expression of total-phosphorylated Erk1/2 was not affected. Similarly, IL-17A activated Akt within 7 min. The phosphorylation of Akt reached a maximum at 15 min and no further increase was observed at later time points. The levels of total Akt remained generally constant. Thus IL-17A activates Erk1/2, p38, and Akt in ARPE-19 cells.

### Effects of signaling inhibitors on CXCL8, CCL2, and IL-6 production induced by IL-17A

**Figure 2:**
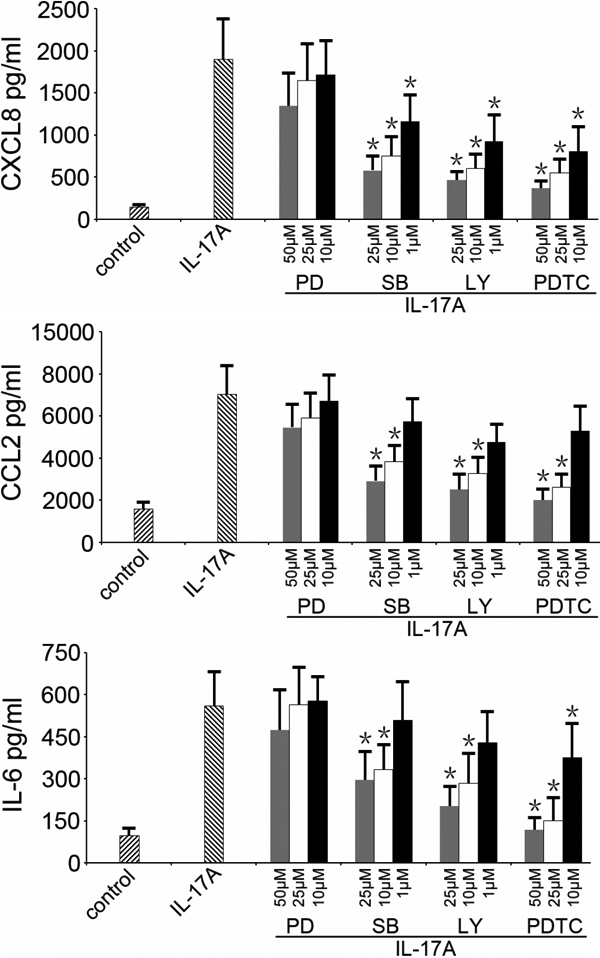
Effects of signaling inhibitors on IL-17A-induced CXCL8, CCL2, and IL-6 production in ARPE-19 cells. Cells were pretreated with PD98059 (PD), SB203580 (SB), LY29400 (LY) or PDTC at the indicated concentrations for 2 h, followed by incubation with or without 100 ng/ml IL-17A for 24 h. Secreted cytokines in supernatants were determined by ELISA. Values are expressed as the mean±SD of four independent experiments. *p<0.05, compared with the values in the absence of inhibitors.

**Figure 3:**
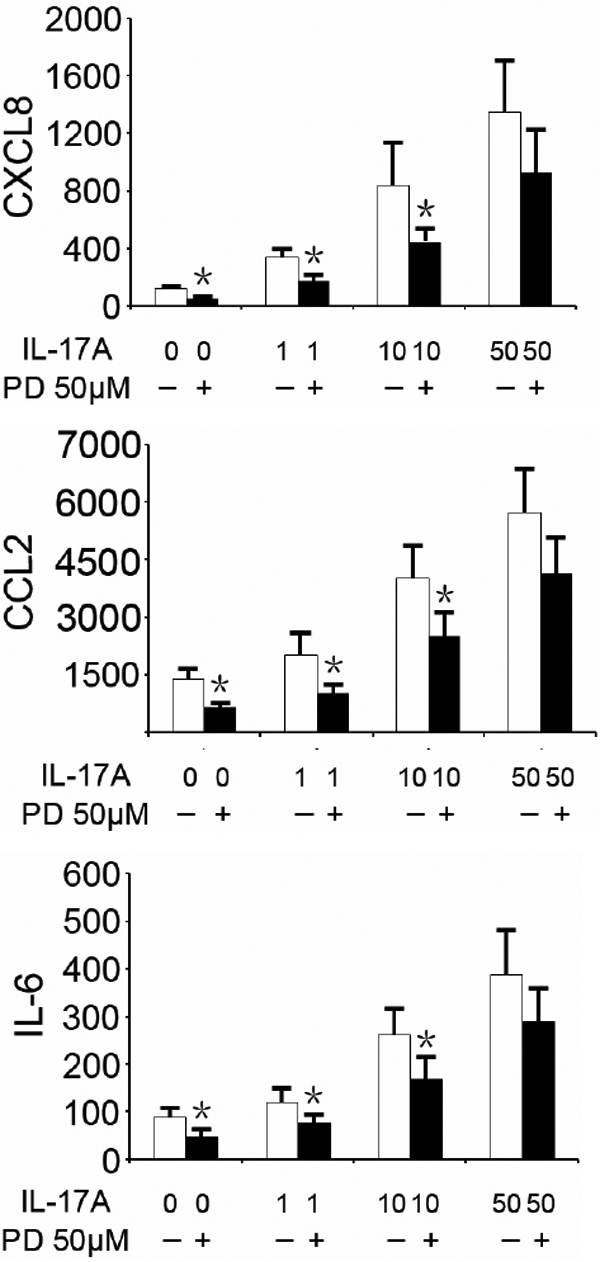
Effects of PD98059 on the expression of CXCL8, CCL2, and IL-6 in ARPE-19 cells simulated with or without different concentration IL-17A. Cells were cultured with or without 50 μΜ PD98059 (PD) for 2 h, followed by incubation with or without 1, 10, and 50 ng/ml IL-17A for 24 h. Secreted cytokines in supernatants were determined by ELISA. Values are expressed as the mean±SD of four independent experiments. *p<0.05, compared with the values in the absence of inhibitors.

To confirm the role of Erk1/2, p38MAPK, Akt, and NF-κB activation in the production of CXCL8, CCL2, and IL-6 by ARPE-19 cells stimulated with 100 ng/ml IL-17A, we investigated the effect of Erk1/2, p38MAPK, Akt, and NF-κB inhibitors. As shown in Figure 2, IL-17A caused a 13.1, 4.5, and 5.9 fold secretion of CXCL8, CCL2, and IL-6 over basal levels, which was considered as 100%. SB203580, an inhibitor of p38MAPK, decreased IL-17A-induced CXCL8, CCL2, and IL-6 production in a dose-dependent manner, resulting in levels of 69±8%, 59±7%, and 47±16%, respectively. Similarly, LY294002 and PDTC, inhibitors of PI3K/Akt and NF-κB also dose-dependently inhibited the expression of CXCL8, CCL2, and IL-6. The levels following incubation with LY29400 were 75±5%, 64±12%, and 65±7%. The maximum levels following PDTC were 80±7%, 70±10%, and 78±8%. The aforementioned inhibitors also markedly repress the basal secretion of CXCL8, CCL2, and IL-6 by ARPE-19 cells in the absence of IL-17A (data not shown). PD98059, an inhibitor of Erk1/2 did not affect the production of the three tested inflammatory mediators when using a dose of 100 ng/ml of IL-17A. A separate experiment using lower concentrations of IL17A showed that PD98059 was able to inhibit the production of these mediators when the concentration of IL-17A used to stimulate the cells was lower than 50 ng/ml (Figure 3).

## Discussion

In this study, we demonstrated that IL-17A is able to enhance the phosphorylation of Erk1/2, p38MAPK, and Akt in RPE cells. The expression of IL-17A-induced CXCL8, CCL2, and IL-6 was decreased in these cells by SB203580, LY294002, and PDTC, inhibitors of p38MAPK, PI3K-Akt, and NF-κB, respectively, in a concentration-dependent manner. We also found PD98059, the inhibitor of Erk1/2 altered the expression of IL-17A-induced inflammatory mediators, depending on the concentration of IL-17A. This study is a continuation of a previous study to determine the intracellular mechanisms of IL-17A-mediated production of CXCL8, CCL2, and IL-6 by RPE cells [13].

Accumulating evidence shows that MAPK activation is an important signaling event in the response of RPE cells to proinflammatory cytokines such as IL-1β and tumor necrosis factor (TNF)-α [18]. We found that IL-17A induces phosphorylation of Erk1/2 and p38 MAPK in RPE cells, which is in accordance with earlier findings in articular chondrocytes [19] and cardiac fibroblasts [20]. We also showed that PD98059 and SB203580, specific inhibitors of Erk1/2 and p38MAPK, reduced IL-17A-induced CXCL8, CCL2, and IL-6 production by RPE cells, indicating that Erk1/2 and p38MAPK are involved in this process. These findings are also in accordance with earlier findings using lung microvascular endothelial cells [14], mesangial cells [15], and human pancreatic periacinar myofibroblasts [16]. It is worthwhile to point out that the PD98059 mediated inhibition of the release of the tested three inflammatory mediators was lost when using higher concentrations of IL-17A. The latter observation is consistent with earlier findings whereby PD98059 was also not able to inhibit IL-17A mediated induction of *CXCL8* mRNA production by fibroblast-like synoviocytes [21]. These data suggest that Erk1/2 may not be the major intracellular molecular signaling pathway regulating the IL-17A-mediated production of CXCL8, CCL2, and IL-6 in non-immune type cells such as the RPE cell or synoviocyte.

It has previously been shown that PI3K and its downstream mediator Akt regulate the production of inflammatory mediators in response to proinflammatory cytokines [22,23]. Akt is activated by a variety of cell surface receptors that when stimulated induce the activation of PI3K. PI3K produces phosphatidylinositol 3, 4, 5 triphosphates (PIP3) which in turn activates Akt. In the present study, we demonstrated that activation of the PI3K-Akt pathway by IL-17A is necessary for CXCL8, CCL2, and IL-6 protein full expression in RPE cells. These results are consistent with other findings in human airway epithelial cells [17], fibroblasts and macrophages obtained from rheumatoid arthritis synovial tissue [17,24]. In contrast to our data, the PI3K/Akt pathway was only involved in the expression of CCL2, not CXCL8 in human RPE following stimulation by IL-1β or TNF-α [25]. The discrepancy may be, at least partially, explained by the nature of the stimulus [25].

The activation of transcriptional factor NF-κB plays a central role in the induction of inflammatory mediators. The promoter regions of the human *CXCL8*, *CCL2*, and *IL-6* genes have been cloned and have been shown to contain consensus binding motifs for NF-κB [26]. Our results showed that PDTC, an inhibitor of NF-κB, significantly inhibited IL-17-induced CXCL8, CCL2, and IL-6 production in RPE cells,suggesting that NF-κB activation was responsible for the expression of these inflammatory mediators. Similar results have been observed using a variety of other cell types [16,17,27].

In conclusion, we have demonstrated that p38MAPK, PI3K-Akt, and the transcription factor NF-κB are involved in IL-17-induced CXCL8, CCL2, and IL-6 release in ARPE-19 cells. Knowledge concerning the mechanisms whereby IL-17A regulates the secretion of inflammatory mediators by RPE cells may increase our insight in the pathogenesis of posterior uveitis and delineating the transactivation mechanisms may help to identify new therapeutic targets.
